# Effectiveness of Disulfiram as Adjunct to Addiction‐Focused Treatment for Persons With Severe Alcohol Use Disorder

**DOI:** 10.1111/adb.70035

**Published:** 2025-04-22

**Authors:** Max Schallenberg, Diana Vogel‐Blaschka, Maik Spreer, Julia Göstl, Johannes Petzold, Maximilian Pilhatsch

**Affiliations:** ^1^ Department of Psychiatry and Psychotherapy University Hospital, Technische Universität Dresden Dresden Germany; ^2^ Clinic for Psychiatry and Psychotherapy Elblandklinikum Radebeul Radebeul Germany

**Keywords:** alcohol dependence therapy, alcohol use disorder, Antabus, craving, disulfiram

## Abstract

The consumption of alcohol affects 400 million people worldwide, where it is responsible for 7% of deaths. Treatment success rates in this field remain limited. Only 15% of those who need treatment get it. Despite treatment, alcohol intake reoccurs in up to 90% of the cases. The use of disulfiram in preventing alcohol reoccurrence is attributed to its unique mechanism of action as an aversive agent, which causes the patient to experience unpleasant physical symptoms when they consume alcohol. The objective of this study is to confirm and illustrate the efficacy of disulfiram in combination with non‐pharmacological intervention for persons with severe AUD. Clinical data from 45 patients of an outpatient treatment programme, including the application of disulfiram (2011–2023) were analysed to assess abstinence rates, craving impact, and demographic factors. Moreover, our analyses aimed to identify predictors and moderators of continuous abstinence duration. The study cohort comprised patients with severe AUD and high rates of comorbidities, the majority of which were affective disorders. During treatment, 50% of patients remained abstinent for at least 1 year. No significant differences were identified in craving, sex or comorbidities compared with those who experienced a return to substance use after treatment initiation. Disulfiram underlined its efficacy and tolerability as an adjunct to addiction‐focused treatment in a typical clinical cohort of patients severely affected by AUD. Moreover, our analyses align with previous research indicating that disulfiram appears to allow patients with AUD to resist craving episodes, therefore avoiding impulsive reoccurrences of alcohol intake.

## Graphical abstract

Disulfiram is experiencing a renaissance in the treatment of persons with severe alcohol use disorder due to its unique mechanism of action on the one hand and the lack of effective pharmacotherapeutic innovations on the other. Our study supports the efficacy and tolerability of disulfiram when prescribed as an adjunct to addiction‐focused treatment. Almost all of the 45 severely and mostly comorbidly affected individuals studied benefited from this therapeutic approach, with 50% remaining abstinent for at least 1 year.

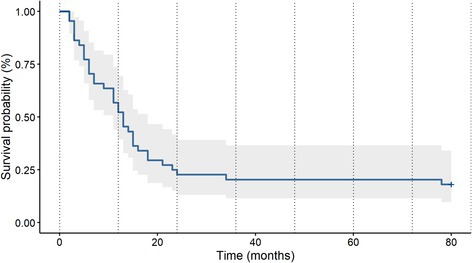

**Abbreviations**
ADR: alcohol disulfiram reaction
AUD: alcohol use disorder
DALY: disability‐adjusted life years
EtG: ethyl glucuronide
ICD: International Classification of Diseases
MBRP: mindfulness‐based relapse prevention
NAP: Network for Alcohol‐Aversive Pharmacotherapy
SOP: standard operation procedure

## 1 Introduction

Alcohol consumption is a serious global issue, affecting approximately 400 million people, or 7% of the world's population, primarily those aged 15 years and older. Of these, over 200 million live with alcohol use disorder (AUD). Alcohol consumption was responsible for 7% of all deaths among people 69 years of age and younger [1]. The effects of AUD are multifaceted and refer to at least 35 different physical and psychological sequelae, such as depression and anxiety [2, 3]. Persons with AUD are susceptible to obstacles impeding their access to healthcare, education, employment opportunities and the process of reintegration into society. In some instances, the influence of socioeconomic challenges may play a role in this. Children of parents with AUD are more likely to show behavioural problems and have an increased risk of developing an addiction themselves [4, 5].

In 2019, alcohol‐related harm constituted a significant proportion of the global burden of disease and injury. It was responsible for 115.9 million DALYs, accounting for 4.6% of all DALYs that year [1].

The course of AUD is variable and encompasses episodes of excessive alcohol consumption, as well as periods of controlled intake or abstinence [3, 6]. A combination of medical treatment, psychological support and social rehabilitation is a crucial component of successful, long‐term, therapeutic interventions.

Notwithstanding the considerable body of evidence on the treatment of AUD and the numerous studies that have been conducted in this field, the success rate of therapeutic interventions remains limited. Firstly, there is a significant gap in the number of individuals having access to addiction support services, with only approximately 15% of those requiring treatment being able to do so [7]. Secondly, reoccurrence is a significant challenge in the recovery process for individuals with AUD. Reoccurrence rates vary considerably, with up to 90% experiencing at least one setback over a four‐year period. The chance of a full reoccurrence depends on various factors, including how it is defined and the person's situation. One‐year reoccurrence rates are 40%–60%, with the highest risk in the first 3 months of abstinence. After 5 years of sustained abstinence, the reoccurrence rate was less than 15%. These figures highlight the need for tailored interventions and ongoing support [8, 9, 10, 11, 12]. From a global perspective, only a minority of approximately 17% of individuals with AUD receive specific therapeutic interventions. Only a subset of the aforementioned population of 17%, amounting to approximately 3%, has access to specific pharmacological treatments [13].

It is widely acknowledged that craving represents a key phenomenon, often preceding substance intake [14].

Considering the limited efficacy of existing treatment modalities, there has been an increase in the utilization of disulfiram as a pivotal component of multimodal treatment regimens over the past decade [15]. Disulfiram is regarded as the earliest and most pioneering pharmaceutical agent for preventing the reoccurrence of alcohol intake [16]. The efficacy of disulfiram has been demonstrated to be superior to that of naltrexone and acamprosate [17]. The available evidence indicates that disulfiram, when used continuously under supervision and in conjunction with supportive psychotherapy, is more effective than standard treatments, such as oral naltrexone, in promoting abstinence. However, while disulfiram's effects are evident in non‐blinded trials due to its distinct aversive mechanism of action, its superiority over other agents is primarily observed in non‐randomized controlled trials (non‐RCTs), where real‐world data can offer valuable insights into its practical application [18, 19].

Disulfiram's distinctive mode of action functions as an aversive agent. Alcohol consumption in the presence of disulfiram therapy results in the development of an adverse reaction known as the alcohol‐disulfiram reaction (ADR). This phenomenon is characterized by various physiological effects, including flushing, hypotension, tachycardia, nausea and vomiting, shortness of breath, headaches, cardiac arrhythmia, as well as cardiac complications, including loss of consciousness [20]. Despite the existence of isolated case reports of fatal outcomes associated with disulfiram therapy, it is notable that such events are exceptional and do not reflect the typical safety profile of the medication when used as prescribed under proper supervision [21, 22].

At present, the administration of disulfiram is confined to a limited number of medical facilities in Germany due to the lack of official approval. Consequently, the establishment of nationwide access to this treatment remains a challenge. However, in 2018, several outpatient psychiatric clinics and registered medical specialists collaborated to establish the ‘Network for Alcohol‐Aversive Pharmacotherapy’ (NAP), with the objective of maintaining the quality of this form of treatment. This study reports data from a selected subset of the NAP, supplementing the recent publication of a quality report, which concentrated primarily on patient characteristics and safety [23].

Here, we assessed the efficacy of supervised disulfiram as an adjunct to addiction‐focused therapy for patients with severe AUD within the psychiatric outpatient clinic of the University Hospital Dresden. More specifically, we assessed the efficacy of our treatment program in preventing reoccurrences in patients with AUD. The primary outcome was the number of patients abstinent for 1 year following initial treatment, along with the mean duration of abstinence. The duration of continuous abstinence was documented during the study period. However, the length of follow‐up varied depending on individual clinical needs. Some patients continued outpatient care due to concomitant psychiatric conditions after completing disulfiram therapy.

The study also looked at how socio‐demographic and clinical factors, such as comorbidities as well as treatment‐related variables, affect the length of abstinence, medical complications, inpatient admissions and pre‐treatment alcohol consumption.

## 2 Methods

### 2.1 Addiction Outpatient Clinic of the University Hospital and Therapy Scheme

The outpatient addiction clinic at the Dresden University Hospital has provided disulfiram treatment since 2009, in accordance with a standardized protocol published elsewhere in more detail [24].

Prior to commencing therapy, physical and psychological evaluations, as well as a series of comprehensive diagnostic tests, were conducted to ascertain the absence of any potential life‐threatening contraindications, such as coronary heart disease or liver cirrhosis, including oesophageal varices. The diagnostic procedures included an exercise electrocardiogram (cardiopulmonary computed tomography scan if indicated), abdominal sonography and laboratory tests. In instances indicative of portal hypertension, gastroscopy was undertaken to identify the presence of oesophageal varices. Furthermore, breath alcohol levels were monitored prior to each dose, and in some cases, ethyl glucuronide (EtG) levels were also determined, thus ensuring ongoing abstinence [23, 24].

Following this assessment of the patient's suitability for therapy and the obtaining of informed consent, the treatment commences with an initial dosage of 0.2–0.4 g per day, under strict alcohol abstinence. The dosage may be increased gradually up to 750 mg three times per week, with 700–3500 mg/week being an efficacious, safe and tolerable dose depending on the individual metabolism status [24]. In conjunction with the administration of disulfiram under continuous medical supervision, patients were offered individual or group psychotherapy, with the objective of providing support for their recovery and addressing the underlying addiction.

Subsequent to the successful implementation of treatment and the establishment of a therapeutic alliance, the frequency of supervised intake could be reduced, potentially to once a week. In the event of an abstinence violation, disulfiram was temporarily discontinued, and a risk–benefit assessment was conducted to determine whether the resumption of disulfiram therapy was advisable. If it was deemed appropriate, the administration of disulfiram at the last administered dosage was reinitiated on the following day, with increased supervision. A comprehensive methodology for the administration of disulfiram is delineated in the SOP conducted by Zimmermann et al. (2021) [23, 24].

### 2.2 Collection of Data

A retrospective data analysis was conducted to investigate the disease courses of all 45 patients who were treated with disulfiram at the outpatient clinic of the University Hospital Dresden between 1 January 2011 and 30 April 2023. This study was based on an evaluation of routine clinical and socio‐biographical data collected as part of the clinical pathways of inpatients and outpatients, where patients were also frequently queried about the presence of craving. If craving was confirmed, it was classified by the participants as mild, moderate, or severe. However, craving was not recorded or documented in a standardized manner. The study was approved by the ethics committee of the TU Dresden (reference number BO‐EK‐188052024).

Statistical analysis of the collected data was evaluated using the statistical software package SPSS Statistics 28.

### 2.3 Aims and Objectives

The principal objective of this study was to assess the efficacy of disulfiram medication as an adjunct in the treatment of AUD, with a particular focus on the number of patients who remained abstinent 1 year following the completion of their initial course of treatment. Furthermore, the mean duration of abstinence (in days) following the initial course of therapy was calculated.

A further objective of this study was to evaluate the influence of socio‐demographic and clinical factors, as well as treatment‐related variables, on the duration of abstinence. A regression analysis was conducted to identify potential predictors of prolonged abstinence, examining factors such as medical complications, the number of inpatient admissions and pre‐treatment alcohol consumption as indicators of alcohol dependence severity. This aspect of the study aimed to ascertain whether a correlation exists between the severity of alcohol dependence and the duration of abstinence achieved.

Furthermore, we examined whether variables such as age, gender, socioeconomic status, reported craving and the presence of comorbid affective disorders influenced the duration of abstinence. This analysis employed the Mann–Whitney *U* test to address this question. To facilitate a more comprehensive understanding of the results of the treatment, a survival analysis was carried out in order to provide a more accurate depiction of the trajectory of abstinence over time.

## 3 Results

### 3.1 Sample

A study was conducted over a twelve‐year period at the outpatient addiction clinic of the University Hospital Dresden, the purpose of which was to assess the efficacy of disulfiram in patients diagnosed with severe AUD according to the International Classification of Diseases (ICD‐10, F10.2) and a treatment‐refractory course. A total of 45 patients were included in the study. At the start of disulfiram treatment, Table 1 presents the data on the subjects' demographic characteristics; Table 2 provides an overview of the subjects' psychiatric and somatic histories; and Table 3 outlines the subjects' alcohol consumption patterns.

**TABLE 1:** Socio‐demographic data.

		Mean	Standard deviation	Number	(%)
Age		52	11		
Sex	Male			30	66.7%
	Female			15	33.3%
Family status	Single			24	53.3%
	Married			8	17.8%
	Divorced			2	4.4%
	Living separately			11	24.5%
Nationality	German			45	100.0%
Highest educational qualification achieved	No school‐leaving certificate or special‐needs school			3	7.0%
	School‐leaving qualification			7	16.3%
	Vocational qualification			27	62.8%
	University degree or higher			6	13.9%
Occupation	Job seeking			12	27.9%
	Employed			20	46.6%
	Retired or incapacitated for work			11	25.6%

**TABLE 2:** Psychiatric and somatic anamnesis.

		Number	(%)
Somatic comorbidities	Metabolic	20	44.4%
	Cardiovascular	11	24.4%
	Gastrointestinal	11	24.4%
	Neurological	10	22.2%
Psychiatric diagnoses	Other substance use disorders (excluding nicotine)	45	100%
	Psychotic disorders	3	6.7%
	Affective disorders	28	62.2%
	Neurotic, stress‐related and somatoform disorders	10	22.2%
	Personality disorders	6	13.3%
	AD(H)D	1	2.2%
Psychiatric medication	Antidepressants	17	37.2%
	Mood stabilizers	2	4.7%
	Antipsychotics	4	9.3%
Family history of AUD	Yes	19	41.5%
	No	26	58.5%
Suicide attempts	At least one	22	48.9%
	Not documented	12	26.7%

**TABLE 3:** Consumption parameters.

		Average	Standard deviation	Number	(%)
Average duration of AUD (years)		14.75	8.9	20	44.4%
Inpatient treatments	Hospitalizations	20.90	22.41		
	Sum of detox and qualified withdrawal treatments	11.88	14.69		
	Addiction rehabilitation	1.93	1.24		
Withdrawal complications	Yes			18	37.2%
	No			27	62.8%
Previous addiction medication	Naltrexone			27	46.6%
	Acamprosate			12	20.7%
	Baclofen			3	5.2%
	Nalmefene			4	6.9%
Age at first manifestation of AUD (years)		34.58	11.79		
Age at first drinking (years)		14.81	2.54		
Illegal substance use	Yes			17	48.1%
	No			28	51.9%
Highest blood alcohol concentration measured before starting disulfiram (g/l)		3.57	0.87		
Average duration of abstinence before starting disulfiram (days)		2.34	2.41		

With regard to comorbidities, the prevalence of metabolic diseases, such as diabetes or hypertriglyceridemia, was 44.4% among patients, cardiovascular and gastrointestinal diseases were each observed in 24.4%, while neurological diseases manifested in 22.2% of the subjects. Prior to the commencement of disulfiram treatment, approximately three‐quarters of the patients exhibited elevated liver values. Regarding psychiatric diagnoses, 62.2% of the test subjects were diagnosed with affective disorders, 22.2% with neurotic and somatoform disorders, 13.3% with personality disorders and 6.7% with schizophrenia. At the commencement of disulfiram treatment, 37.2% of patients were receiving antidepressant medication, 9.3% antipsychotic drugs and 4.7% mood stabilizers. In terms of family history, 41.5% of the participants had at least one parent who was dependent on alcohol. It should be noted that information pertaining to suicide attempts was not available for 48.9% of the participants, whereas 26.7% had made at least one attempt.

The mean age at the initial onset of AUD was 34.6 years, while the mean age at the first instance of drinking was 14.8 years. The average duration of AUD among the subjects was 14.8 years. The mean number of hospitalizations per patient was 20.9, while the mean number of qualified withdrawal treatments, which addressed not only physical symptoms but also incorporated parallel psychotherapy and social therapeutic elements in addition to addiction rehabilitation, was 11.9 and 1.9, respectively. Prior to the commencement of disulfiram treatment, 46.6% of patients were administered naltrexone, 20.7% acamprosate, 5.2% baclofen and 6.9% nalmefene. Of the test subjects, 62.8% exhibited no documented withdrawal complications, whereas 51.9% indicated that they did not engage in the consumption of any additional addictive substances (not considering tobacco and caffeine) alongside alcohol. Among the remaining 48.1%, 25.9% (*n* = 14) reported the use of cannabis, 9.3% (*n* = 5) reported the use of crystal methamphetamine and 7.4% (*n* = 4) reported the use of cocaine. Furthermore, 5.6% (*n* = 3) of the patients were reported to have used amphetamines.

### 3.2 Course of Treatment

**FIGURE 1:**
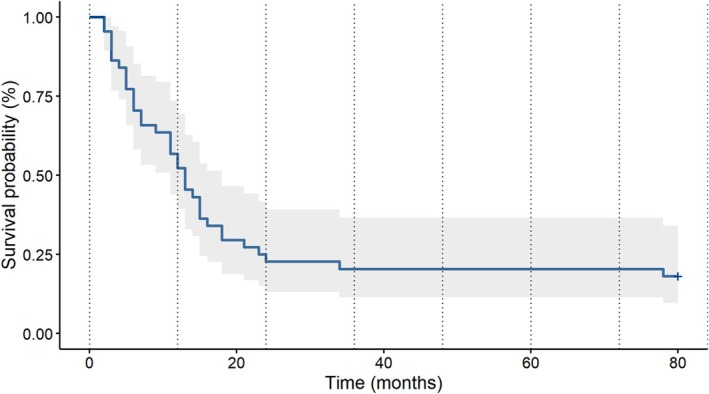
Time to reoccurrence of alcohol intake after initiating disulfiram therapy (grey space is confidence interval).

The interval between admission to the outpatient clinic and the commencement of disulfiram treatment averaged 9.8 months, with the overall treatment duration averaging 43.3 months. The participants were, on average, 45.7 years of age (SD 10.9 years) at the initiation of disulfiram treatment. The mean duration of abstinence following the initial treatment with disulfiram was 16.27 months. A total of 50% of participants remained abstinent for a minimum of 1 year following the initial commencement of disulfiram treatment (Figure 1). A total of 17.8% of the patients did not experience any abstinence violation during disulfiram treatment. Among the remainder of the patients, reoccurrence of alcohol intake manifested as the independent cessation of disulfiram in 44.4% of cases, with no discernible trigger in 26.7% of instances and as an outcome of stressful life events in the remaining 11.5% of cases. During the aforementioned period, the mean number of abstinence violations per patient was 2.9. In the course of disulfiram treatment, 54.5% of the patients reported no craving, while 44.4% exhibited a varying degree of craving (Table 4).

**TABLE 4:** Disulfiram therapy progression parameters.

		Mean	Standard deviation	Number	(%)
Age at start of disulfiram (years)		45.73	10.94		
Abstinence period after first start of disulfiram (months)		16.27	20.10		
Time to start disulfiram after connection to the outpatient clinic (months)		9.84	30.05		
Duration of treatment with disulfiram (months)		43.30	30.05		
Reoccurrence on disulfiram	None				17.8%
	Self‐discontinuation				44.4%
	Without recognizable trigger				26.7%
	Connection with stressful life event				11.5%
Abstinence violations between 01.2018 and 04.2023		2.89	3.17		
Course of therapy	Regular visits but inadequate intake of disulfiram			7	15.6%
	Frequent self‐discontinuation and associated reoccurrence			8	17.8%
	Treatment cancellation			1	2.2%
	Regular visits and intake of disulfiram			29	64.4%
Parallel participation in self‐help groups	Yes			35	77.8%
	No			10	22.2%
Parallel psychotherapy (group or single)	Yes			39	86.7%
	No			6	13.3%
Craving	None			24	54.5%
	Yes			20	44.4%
	Not specified			1	2.2%
Disulfiram‐alcohol reaction	Light			4	8.9%
	Mild			6	13.3%
	Severe			3	6.7%
	Without symptoms			32	71.1%

With regard to medication adherence, 64.4% displayed consistent and reliable intake of their medication as prescribed. They also demonstrated reliable attendance at their scheduled appointments. In comparison, 15.6% of the sample exhibited inconsistent behaviour in at least one of these respects. Of these patients, 17.8% discontinued their medication without professional guidance, resulting in reoccurrence. A 25% attrition rate was observed among patients who experienced their first abstinence violation during disulfiram treatment. This lapse often marked the point at which they were unable to maintain participation in the program, including attending scheduled appointments in the program, highlighting the challenge of sustaining adherence following an initial setback. The remaining patients who had experienced at least one reoccurrence were able to remain in the program for a further 22 months on average.

Psychotherapy (group or single), with a frequency of two to four times a month, was provided as an adjunct to the treatment regimen in 86.7% of cases, while 77.8% of patients participated in self‐help groups.

### 3.3 Interference Statistics

Mann–Whitney *U* tests yielded no significant differences in the abstinent period after the first start of disulfiram treatment between patients with and without reporting craving (*z* = −0.50, *α* = 0.05, *p* = 0.62), between patients with and without a comorbid affective disorder (*z* = *−1.64*, α = 0.05, *p* = 0.10), or between women and men (*z* = *−*1.68, *α* = 0.05, *p* = 0.09). However, no significant differences were found between reoccurrence yes or no (*Z* = −1.51, α = 0.05, *p* = 0.13; Tables 5 and 6).

The multiple linear regression analysis on the duration of abstinence after the first start of disulfiram treatment showed that the number of inpatient detoxifications (Beta = *−*0.27, *t* (3) = *−* 1.65, *p* = 0.11) and the average alcohol consumption before the start of disulfiram therapy (Beta = −0.15, *t* (3) = *−* 0.90, *p* = 0.37) did not have a significant impact, whereas the number of complications during abstinence after the first start of disulfiram treatment (Beta = 0.45, *t* (3) = 2.71, *p* = 0.01) showed a significant impact (Tables 7–9).

The model accounted for 20% of the total variance of the abstinent period after the first start of disulfiram treatment. However, the value of the corrected R‐squared was 0.13, with a *p* value of (0.05), indicating that the model is unable to provide a satisfactory explanation of the data.

## 4 Discussion

The results of our study highlight the potential of disulfiram as an adjunct to addiction‐focused treatment for severe AUD. The efficacy observed in our sample was even more pronounced than that reported in comparable studies, despite the comparatively severe treatment courses prior to the commencement of treatment. As in other studies, disulfiram was well tolerated.

### 4.1 Outcome Variables

With respect to efficacy, the mean duration of abstinence for the patients in the sample was 16 months following the initiation of the treatment program. After 1 year, 50% of participants were still abstinent, which serves to illustrate the potential for the sustained success of treatment programmes utilizing disulfiram. It is important to note, however, that not all instances of non‐adherence to the abstinence requirement resulted in the patient's withdrawal from the therapeutic program [24]. Indeed, many patients who experienced a reoccurrence were able to remain engaged in therapy, which allowed clinicians to continue providing crucial support and interventions that had a positive impact on the course of their addiction treatment.

The maintenance of patients within the therapeutic framework, even in the event of an abstinence violation, is a crucial factor in the promotion of long‐term recovery [25]. This approach recognizes that the reoccurrence of alcohol intake is frequently an inherent aspect of the recovery process and does not necessarily indicate a failure of treatment itself. By facilitating continued engagement, the treatment program reinforced the importance of persistence and adaptability, thereby providing patients with a safety net rather than viewing reoccurrence as a disqualifying event.

Through introspective analysis and discourse with the therapeutic team, a significant number of patients were able to discern the emotional, social and environmental factors that precipitated their reoccurrence. This process facilitated the acquisition of valuable insights into their personal vulnerabilities and the development of more robust coping strategies for future challenges. By addressing these underlying issues, patients were able to reaffirm their commitment to the treatment process [26].

Consequently, the majority of patients decided to continue with the program following an abstinent violation.

In their respective studies, Hochsattel (2016) reported an average abstinence period of 12 months for 24% of their patients [27, 28].

During treatment, 77.8% of the patients participated in self‐help groups, while 86.7% engaged in accompanying psychotherapy. By comparison, Stahlberg (2018) reported a participation rate of only 35.5% in self‐help groups, which suggests that our sample was particularly motivated to utilize a range of therapeutic supports. This indicates that disulfiram might be seen as a final opportunity for effective AUD therapy and patients seek to utilize all available support [27].

The present study observed an average of 0.89 instances of non‐adherence to the prescribed abstinence regimen per patient, with a total of 80 documented instances between January 2018 and April 2023. In contrast, Stahlberg et al. (2018) documented an average of 0.42 reoccurrences per patient [27]. It can be reasonably assumed that the higher rate of abstinence violations in our study overall can be attributed to two outliers in which disulfiram was employed as part of a harm reduction strategy, subsequent to the determination that these patients would derive significant benefit from disulfiram, but complete abstinence was not a feasible outcome [29].

### 4.2 Sample

Addressing socio‐biographical parameters such as sex distribution, average age, educational attainment, professional status and relationship status, any discrepancies from the current data in the field of disulfiram treatment were minimal and isolated [27, 30, 31].

Ehrenreich et al. implemented a meticulously designed programme for patients who missed appointments. Despite these methodological differences, our study observed a substantially higher employment rate (46.6%) compared to the 28% reported by Ehrenreich et al. This difference in sample characteristics [30, 32] might suggest that our cohort might have been more socially and professionally integrated at baseline, which could have positively influenced treatment outcomes. Moreover, our results demonstrated that disulfiram therapy was well tolerated and effective in a naturalistic outpatient setting, supporting its feasibility even outside highly controlled intervention programmes.

Furthermore, while the more intensive approach of Ehrenreich et al. may have provided stronger external reinforcement for abstinence, our findings suggest that structured yet flexible outpatient treatment, including supervised disulfiram administration combined with psychotherapy, can also yield favourable long‐term abstinence rates. This finding is consistent with previous research that highlighted the pivotal roles of treatment adherence and integration into daily life activities, such as employment, in maintaining recovery [33]. Participants can continue working while undergoing treatment.

In conclusion, the present findings corroborate those of Ehrenreich et al. by demonstrating the efficacy of disulfiram‐based treatment not only in highly intensive programmes but also in real‐world outpatient settings, where patients maintain a greater degree of autonomy over their treatment course.

In consideration of the clinical characteristics, the average duration of AUD was observed to be 14.75 years, which aligns with the findings of previous studies in this area. [27, 30, 34]. The average number of previous qualified withdrawal treatments was 11.8. Additionally, the average daily consumption of approximately 280 g of pure alcohol reported per day is comparable to the results of other studies, which underlines the severity of AUD in our sample. Moreover, an average of 1.1 instances of withdrawal complications during the study period, such as seizures, was reported per patient. A review of the literature reveals that psychiatric comorbidities are a common occurrence in individuals with alcohol dependence [35]. The findings of our study indicate that approximately 73.3% of the participants fulfilled criteria of at least one psychiatric comorbidity, the majority (62.2%) of which was an affective disorder. Most patients with comorbid psychiatric disorders received disorder‐specific therapy as part of the multimodal treatment offered by our outpatient clinic. About half of the patients with comorbid affective disorders were treated with an antidepressant. Others had received psychotherapy. This prevalence rate is higher than that reported in other studies, such as that conducted by Stahlberg (2018) [27]. A higher proportion of patients were diagnosed with neurotic, stress, and somatoform disorders (22.2%) compared to other studies, such as those by Hochsattel (2016) and Stahlberg et al. (2018) [27, 30]. It is acknowledged that routine clinical data is likely to underestimate the true prevalence rates. The relatively high rates of psychiatric comorbidities in our sample may be attributed to the prolonged duration of treatment but could also be indicative of a particularly ill sample. The relationship between AUD and other mental health conditions, such as anxiety disorders and those affecting personality and behaviour, was also investigated. These comorbidities are known to have a substantial impact on treatment outcomes, necessitating a differentiated approach to treatment [26].

The mean age of patients admitted to the study was 46 years. This value is slightly higher than the comparative values from previous studies. The mean duration of treatment in our study was 43.34 months, in contrast to the 20 months reported by Stahlberg (2018). A comparison of the dosage of disulfiram used in the present study with that used by Stahlberg et al. reveals a notable difference in the quantity and frequency of administration. The present study used 750 mg of disulfiram three times a week, which is a relatively high dose. In contrast, Stahlberg et al. administered 125 to 250 mg daily. The difference in how disulfiram was administered between the two studies reflects a key behavioural aspect of adherence to treatment. In Stahlberg's study, patients had to make a deliberate decision to take disulfiram daily, creating a routine that required consistent commitment. This approach emphasizes personal accountability and discipline, which may help reinforce the patient's conscious decision to remain abstinent. However, it also increases the risk of missed doses, as daily adherence can be challenging for some individuals, especially without supervision.

In contrast, our study implemented a dosing schedule of 750 mg three times per week, reducing the frequency of decision‐making required. This strategy may ease the burden on patients and enhance adherence, especially in supervised settings. The patient had taken the decision for abstinence only three times per week. Supervised administration, combined with the reduced frequency, likely minimized opportunities for non‐compliance, ensuring a more consistent therapeutic effect. Research suggests that simplifying treatment regimens can improve adherence, particularly in chronic conditions like AUD [36].

Additionally, the three‐times‐per‐week dosing schedule aligns with disulfiram's pharmacokinetics [16].

Ultimately, while both approaches have merits, the less frequent yet higher‐dose regimen used in our study might offer a practical advantage in terms of adherence, especially in populations where daily medication routines pose challenges.

This may have had a positive effect on the length of time over which abstinence was maintained [27].

In terms of tolerability, our findings indicate that the number of patients who discontinued disulfiram therapy as a consequence of adverse effects was 1 in 45. Disulfiram represents a key medication in the treatment of AUD, as evidenced by both Stahlberg's research and our own findings. These studies underscore the efficacy of the medication in assisting patients in maintaining abstinence, even in instances where conventional approaches have proven ineffective [27].

### 4.3 Additional Parameters

The findings of our study indicate that the severity of AUD (as indicated by the number of previous inpatient stays, history of unsuccessful quit attempts and withdrawal complications) had no significant impact on the length of abstinence following the commencement of disulfiram treatment. The number of inpatient stays had no impact on the efficacy of the disulfiram intervention, as was also demonstrated in the study by Hochsattel (2016) [30]. The proposed therapy with disulfiram might represent a final choice that is currently under consideration [27, 28]. It is established that the occurrence of affective disorders during and after a course of disulfiram treatment is associated with a complex course of treatment and an increased risk of reoccurrence. Depressive disorders have been demonstrated to elevate the likelihood of chronic impairment, particularly when occurring concurrently with unmanaged AUD [37, 38, 39].

The literature presents evidence supporting the superior efficacy of disulfiram as an adjunct to addiction‐focused therapy compared to other pharmacological agents for preventing reoccurrence in patients with AUD and concomitant affective disorder [40, 41]. This assumption is, at the very least, tentatively supported by the results of our study (Mann–Whitney‐*U*‐Test *p* = 0.*05*, Table S5). We postulate that disulfiram may prove especially efficacious in the treatment of affective disorders, given that these patients tend to exhibit a greater proclivity towards anxious avoidance rather than impulsivity.

Interestingly, in our sample, reporting of craving during the therapy did not correlate with subsequent alcohol intake. This result corroborates previous research, namely, that abstinence rates under disulfiram are not influenced by craving over time [42]. This finding might be worthy of further investigation to determine whether disulfiram may be capable of disrupting the established link [43, 44, 45, 46] between craving and alcohol intake. It is possible that psychological factors, such as aversive learning mechanisms or extinction of an action‐reward association, specifically between craving and alcohol intake might be involved. In order to investigate the relationship described in future studies, it would be necessary to implement a modified study design at least using detailed craving assessments and data on craving and its connection to alcohol use before disulfiram was initiated.

### 4.4 Limitations

The absence of a control group renders the study unsuitable for the purpose of determining effects that are specific to our treatment program. The patients in our sample were severely ill, comparable to a typical treatment sample in a care clinic. However, they exhibited a high motivation for abstinence, which may not be representative of all patients with severe AUD. The relatively modest sample size elevates the probability of both false positive and false negative outcomes. The assessment of craving was not conducted in a systematic manner, nor was it carried out with the use of specific instruments. In consideration of all these limitations, it is therefore recommended that the study results be interpreted with caution and that they be confirmed in larger, prospective controlled studies using standardized craving assessments over time.

## 5 Conclusion

To conclude, our study demonstrates the efficacy of disulfiram as an adjunctive treatment for severe and refractory cases of AUD. Our real‐world findings also lend greater support to the notion of attempting a course of disulfiram treatment in the presence of affective comorbidities. Furthermore, the tolerability of disulfiram underscores a favourable risk–benefit analysis, provided that certain conditions and modalities are adhered to [23, 24].

## Ethics Statement

The study was approved by the ethics committee of the TU Dresden (reference number BO‐EK‐188052024).

## Consent

The consent is readily available.

## Conflicts of Interest

The authors declare no conflicts of interest.

## Supporting information


**Data S1.** Supporting Information

## Data Availability

The datasets used and/or analysed during the current study are available from the corresponding author on reasonable request.
