# Supplementary material for: Effectiveness of Disulfiram as Adjunct to Addiction‐Focused Treatment for Persons With Severe Alcohol Use Disorder
Source: Addict Biol. 2025 Apr 22;30(4):e70035. doi: 10.1111/adb.70035 (PMC12012990; doi:10.1111/adb.70035)
Supplement: Supplementary file 1 — Data S1. Supporting Information [file ADB-30-e70035-s001.docx]

# Supplement

Table 5: Mann-Whitney-U-Test craving, gender, affective disorder

|  | a | b | c | d | e | f |  |
| --- | --- | --- | --- | --- | --- | --- | --- |
|  | Abstinence period after first start of disulfiram therapy in months | Abstinence period after first start of disulfiram therapy in months | Abstinence period after first start of disulfiram therapy in months | Abstinence period after first start of disulfiram therapy in months | Abstinence period after first start of disulfiram therapy in months | Abstinence period after first start of disulfiram therapy in months |  |
| Mann-Whitney U | 209.50 | 143.50 | 161.50 | 72 | 220 | 97 |  |
| Z | -.50 | -1.68 | -1.64 |  |  | |  |
| Sig. (1-tailed) | .62 | .09 | .050 | 0.790 | 0.613 | 0.588 |  |
|  |  |  |  |  |  |  |  |

| 1. Grouping Variable: Craving yes or no 2. Grouping Variable: Gender (women/men) 3. Grouping Variable: Affective disorders yes or no 4. Grouping Variable: MDD yes or no 5. Grouping Variable: Antidepressant yes or no 6. Grouping Variable: Antidepressant by patients with major depression yes or no |
| --- |

Table 6: Mann-Whitney-U-Test reoccurrence

|  | Reoccurrence | |
| --- | --- | --- |
| Mann-Whitney U | 177.00 | |
| Z | -1.51 | |
| Sig. (2-tailed) | .13 | |
| a. Grouping Variable: Reoccurrence Yes or No | |  |

Table 7: Model Summary

| Model |  | R Square |  | Std. Error of the Estimate |
| --- | --- | --- | --- | --- |
| 1 |  | .2 |  | 16.16 |

| Predictors: (Constant), Number of complications, sum of inpatient detox and qualified withdrawal treatment, average consumption before starting disulfiram treatment in grams of alcohol |
| --- |

Table 8: ANOVA

|  | | Sum of squares | |  | | Mean square | | F | |
| --- | --- | --- | --- | --- | --- | --- | --- | --- | --- |
| Regression |  | | 3 | |  | | 2.87 | |  |

|  | | Sig. |
| --- | --- | --- |
|  | Regression | .05^b^ |
|  |  |  |
|  |  |  |

| Dependent Variable: Abstinence period after first start of disulfiram therapy in months |
| --- |
| Predictors: (Constant), number of withdrawal complications, sums of inpatient detox and qualified withdrawal treatments, average consumption before starting disulfiram treatment in grams of alcohol |

|  | | Unstandardized Coefficients | | Standardized Coefficients |
| --- | --- | --- | --- | --- |
|  |  | B | Std. Error | Beta |
| 1 | (Constant) | 18.646 | 5.556 |  |
|  | Number of inpatient detoxifications | -.305 | .185 | -.267 |
|  | Average consumption before starting disulfiram treatment in grams of alcohol | -.016 | .018 | -.149 |
|  | Number of withdrawal complications | 3.323 | 1.225 | .453 |

Table 9: Regression

|  | | | | |
| --- | --- | --- | --- | --- |
|  | | t | Sig. | Correlations |
|  |  |  |  | Zero-order |
|  | (Constant) | 3.36 | .00 |  |
|  | Sum of inpatient detox and qualified withdrawal treatments | -1.65 | .11 | -.17 |
|  | Average consumption before starting disulfiram treatment in grams of alcohol | -.90 | .37 | -.06 |
|  | Number of withdrawal complications | 2.71 | .01 | .32 |

Dependent Variable: Abstinence period after first start of disulfiram therapy in months
